# Recurrent anti-TIF1γ-positive dermatomyositis coexisting with postoperative parotid lymphoepithelial carcinoma: a case report with pathogenesis analysis

**DOI:** 10.3389/fimmu.2026.1748650

**Published:** 2026-02-16

**Authors:** Xiaoguang Cui, Kaihong Ye, Hong Wang, Yali Kang, Junqiao Feng, Yameng Wei, Nan Xu, Fuqian Lei, Shan Wang, Rick F. Thorne, Xueyi Li, Ting La

**Affiliations:** 1 National-Local Joint Engineering Research Center of Biodiagnosis & Biotherapy, The Second Affiliated Hospital of Xi’an Jiaotong University, Xi’an, Shaanxi, China; 2 Department of Rheumatology and Immunology, The Second Affiliated Hospital of Xi’an Jiaotong University, Xi’an, Shaanxi, China; 3 Translational Research Institute of Henan Provincial People’s Hospital and People’s Hospital of Zhengzhou University, Tianjian Laboratory of Advanced Biomedical Sciences, Academy of Medical Sciences, Zhengzhou University, Zhengzhou, Henan, China; 4 Precision Medical Research Institute, The Second Affiliated Hospital of Xi’an Jiaotong University, Xi’an, Shaanxi, China; 5 Department of Clinical Laboratory, The Second Affiliated Hospital of Xi’an Jiaotong University, Xi’an, China; 6 Department of Nephrology, The Second Affiliated Hospital of Xi’an Jiaotong University, Xi’an, China

**Keywords:** anti-TIF1γ, case report, dermatomyositis, parotid lymphoepithelial carcinoma, scRNA-seq

## Abstract

**Background:**

Anti-TIF1γ-positive dermatomyositis (DM) is a classic paraneoplastic syndrome in adults, but its coexisting with lymphoepithelial carcinoma (LEC) of the parotid gland is exceptionally rare. This rarity poses significant challenges for clinical management.

**Objective:**

To report a unique case of parotid LEC emerging three years after a diagnosis of anti-TIF1γ-positive DM, followed by a post-oncologic DM recurrence. We aimed to investigate the underlying immunopathogenesis through peripheral blood mononuclear cell (PBMC) analysis and genetic profiling.

**Case presentation:**

A 28-year-old male presented with anti-TIF1γ-positive DM. Three years later, he developed parotid LEC, with Epstein-Barr virus (EBV) detected in both tumor tissue and serology. He was treated with surgical resection and adjuvant therapy, achieving a near-complete oncologic response. However, DM recurred eight months after the cancer diagnosis. Initial cyclophosphamide treatment was effective, but its withdrawal led to relapse; subsequent therapies with methotrexate and tofacitinib provided minimal benefit.

**Results:**

PBMC analysis during the DM recurrence revealed a highly active B-cell population and a reduction in cytotoxic cells. This B-cell expansion subsequently decreased 10 months later, suggesting a delayed effect of the documented EBV activation. Germline genotyping identified a panel of deleterious germline mutations in immune regulation genes, including a variant in *CR2* (rs367567954), which encodes a receptor for EBV on B cells and may contribute to their aberrant activation.

**Conclusion:**

This case illustrates that refractory anti-TIF1γ-DM can persist even after the associated malignancy is well controlled and underscore the need for long-term vigilance and personalized management strategies in paraneoplastic DM.

## Introduction

Lymphoepithelial carcinoma (LEC) is a rare and aggressive malignancy defined histologically by nests of malignant epithelial cells infiltrated by a prominent lymphoid stroma (1). It is strongly associated with EBV (2, 3), with a particularly high incidence in Southern Chinese and Inuit populations (4, 5). Parotid LEC constitutes less than 1% of all salivary gland tumors, and consequently, current knowledge is largely confined to case reports and small series (6–10). The oncogenic potential of EBV in LEC is primarily driven by its latent infection state, during which viral proteins such as latent membrane proteins 1 and 2 (LMP1 and LMP2) promote tumorigenesis by enhancing cell survival, proliferation, and inhibiting apoptosis (11–14). Given this role, EBV is considered a classical oncovirus, and the quantitative analysis of circulating EBV DNA in plasma has become a highly sensitive tool for staging, prognostication, and monitoring of LEC (15, 16).

Dermatomyositis (DM) is an idiopathic autoimmune inflammatory disorder characterized by distinctive skin rashes and progressive muscle weakness (17). It is systemically involved and carries a well-established increased risk of visceral malignancies (18–20). This risk is most pronounced in the subset of adult patients with anti-transcription intermediary factor 1-gamma (anti-TIF1γ) antibodies, where the reported cancer association ranges from 50% to 80% (19, 21). Common associated malignancies include ovarian, lung, breast, pancreatic, gastric, colorectal, and nasopharyngeal carcinomas (the latter being especially prevalent in Asian cohorts) (17, 19, 22). Advanced age is an established risk factor for malignancy in patients with dermatomyositis (21), and the prognosis of dermatomyositis is generally favorable upon effective control of the underlying tumor (23–26). However, anti-TIF1γ-positive DM is seldom reported in association with LEC (27), and to the best of our knowledge, no case of parotid LEC co-occurring with DM has been documented in the literature.

We report a rare case of recurrent anti-TIF1γ-positive DM in a young patient with EBV-associated LEC of the parotid gland. Despite successful tumor control with surgical resection and adjuvant therapy, the patient experienced a DM recurrence two months later. The recurrent DM initially responded to cyclophosphamide and low-dose methylprednisolone but proved refractory to subsequent methotrexate and tofacitinib maintenance therapy after reaching the maximum cumulative dose of cyclophosphamide. To investigate the pathogenesis, we performed single-cell RNA sequencing (scRNA-seq) to profile the peripheral blood mononuclear cell (PBMC) atlas at the baseline of DM recurrence, alongside germline mutation testing via next-generation sequencing (NGS).

## Results

### Clinical presentation

In June 2021, a 28-year-old male presented to our hospital with a triad of diffuse facial erythema, progressive proximal limb weakness, and significant fatigue. He reported no systemic symptoms such as fever or weight loss. Physical examination revealed characteristic heliotrope rash and Gottron’s papules. Laboratory investigations were significant for markedly elevated muscle enzymes: creatine kinase (CK) 1428 U/L (ref: 50–310 for male), CK-MB 38 U/L (ref: 0-19), lactate dehydrogenase (LDH) 350 IU/L (ref: 120-250), aspartate aminotransferase (AST) 71 IU/L (ref: 13-35), and myoglobin 146.4 ng/mL (ref: 11.1-57.7). Electromyography demonstrated a myopathic pattern without denervation potential. Serological testing was positive for the anti-TIF1γ antibody (++; Euroline Autoimmune Inflammatory Myopathies 16 Ag (IgG) Profile, Euroimmun, Lübeck, Germany). A subsequent muscle biopsy confirmed the diagnosis of DM. Given the positive serum anti-TIF1γ antibody result, a whole-body CT scan, chosen for its lower cost compared to PET/CT, was performed for cancer screening. No tumor was detected. Additionally, the patient indicated that there is no family history of any specific health issues. The patient then received an 8-day hospitalization involved with daily intravenous methylprednisolone (60 mg). Following discharge, the rash had improved but persisted. Thus, an outpatient oral steroid taper was instituted, beginning at 40 mg daily and decreasing by 5 mg every two weeks until discontinuation. The patient was subsequently stable.

**Figure 1:**
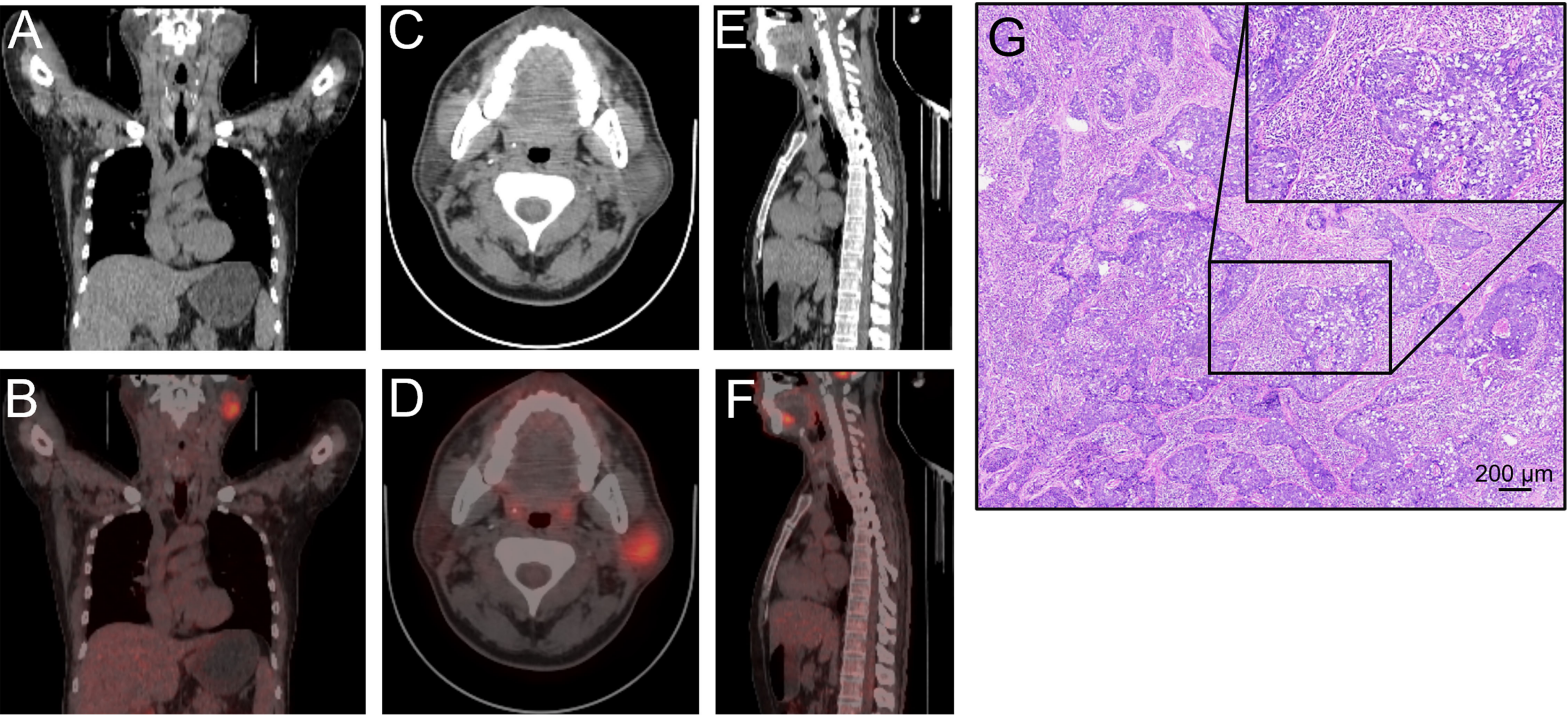
Initial clinical presentation of the parotid lymphoepithelial carcinoma (LEC). **(A–F)** Body anterior view (left, A and B), head horizontal view (middle, C and D) and body lateral view (right, E and F) of PET/CT findings of LEC in the case. **(G)** Routine hematoxylin and eosin (H&E) staining of the LEC tissue. Scale bar 200 μm.

**Figure 2:**
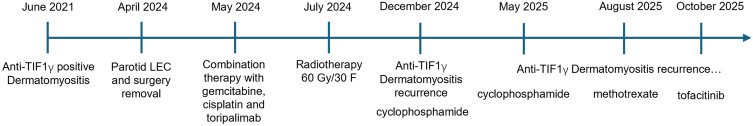
The disease progression and treatment timeline for the patient.

In April 2024, the patient presented to an outside oncology department with left cheek swelling and numbness. Concurrently, a rash was noted on the face and neck. A PET/CT scan revealed a hypermetabolic mass in the left parotid gland, suggestive of malignancy, along with concurrent enlargement of regional lymph nodes (**Figures 1A**–**F**). Subsequent MRI confirmed the presence of bilateral parotid space-occupying lesions. The patient subsequently underwent an extended parotidectomy with suprahyoid lymph node dissection and a right mandibular lymph node biopsy. Histopathological examination confirmed the diagnosis of lymphoepithelial carcinoma, supported by the following findings: H&E staining (**Figure 1G**) showed massive lymphocytic infiltration; immunohistochemistry was positive for CK5/6 and p63, with a Ki-67 proliferation index of 60% in tumor cells; and *in situ* hybridization (ISH) was positive for Epstein-Barr virus-encoded small RNAs (EBER). The diagnosis was further corroborated by the detection of high levels of EBV DNA in peripheral blood. Following surgery, the patient underwent three cycles of adjuvant therapy with gemcitabine (1.8 g/day), cisplatin (50 mg/day), and toripalimab (240 mg), all administered on a 3-week cycle. No severe adverse events were observed during this combination therapy. This was followed by consolidative radiotherapy with a total dose of 60 Gy in 30 fractions. By the completion of systemic therapy on September 15, 2024, the patient had achieved a near-complete response, corroborated by the clearance of EBV DNA to undetectable levels in peripheral blood (**Figure 2**).

Two months after completing systemic oncological treatment (December 2024), the patient developed a pronounced facial rash, recurrent proximal muscle weakness and myalgia. Respiratory symptoms included shortness of breath and a productive cough with yellow sputum. There was no reported fever or dysphagia. He was readmitted to our hospital for comprehensive evaluation. Physical examination revealed classic dermatomyositis findings: symmetrical, edematous, heliotrope erythema on the eyelids; a diffuse, V-shaped erythema on the chest and neck; and symmetrical, dark-purplish Gottron’s papules on the upper extremities. Proximal muscle strength was graded 4/5 with notable tenderness. SARS-CoV-2 nucleic acid was detected in a throat swab sample. Echocardiography revealed no evidence of cardiac disease. Laboratory studies showed a recurrence of elevated muscle enzymes: CK 335 U/L, CK-MB 26 U/L, LDH 357 IU/L, and AST 47 IU/L. Immunological testing was positive for antinuclear antibody (ANA) at a titer of 1:320 and anti-TIF1γ antibody at a titer of 1:30 (cell-based assay, RareDiagnostics, Xi’an, China). Furthermore, the absence of both rheumatoid arthritis (RA)-specific joint symptoms and systemic lupus erythematosus (SLE)-related mucocutaneous findings allowed for the exclusion of these diagnoses. Considering the temporal association with oncologic therapy and the classic clinical and serological profile, a diagnosis of recurrent DM was established (**Figure 2**).

In response to the DM flare, the immunosuppressive regimen was modified to intravenous cyclophosphamide (0.6 g every 2 weeks) for its dual autoimmune and oncological benefits, alongside a reduced dose of methylprednisolone (20 mg initially). During a 6-day hospitalization, the patient’s muscle weakness resolved and enzyme levels normalized. He was discharged on a maintenance methylprednisolone taper, which was later discontinued as his dysphagia and myalgia remained in remission; clinical focus shifted to the more critical paraneoplastic malignancy. A residual rash was present but non-bothersome (**Figure 2**).

Five months later, the rash worsened significantly, requiring re-initiation of cyclophosphamide. After six cycles reached the maximum cumulative dose when his BMI was 18.1, maintenance therapy was switched to subcutaneous methotrexate (10 mg/week), which was ineffective. Tofacitinib was then initiated for rash control, acknowledging the potential malignancy risk. Throughout this period, quarterly head/neck MRI surveillance confirmed the LEC remained stable (**Figure 2**).

### Single-cell transcriptome analysis of the PBMCs

**Figure 3:**
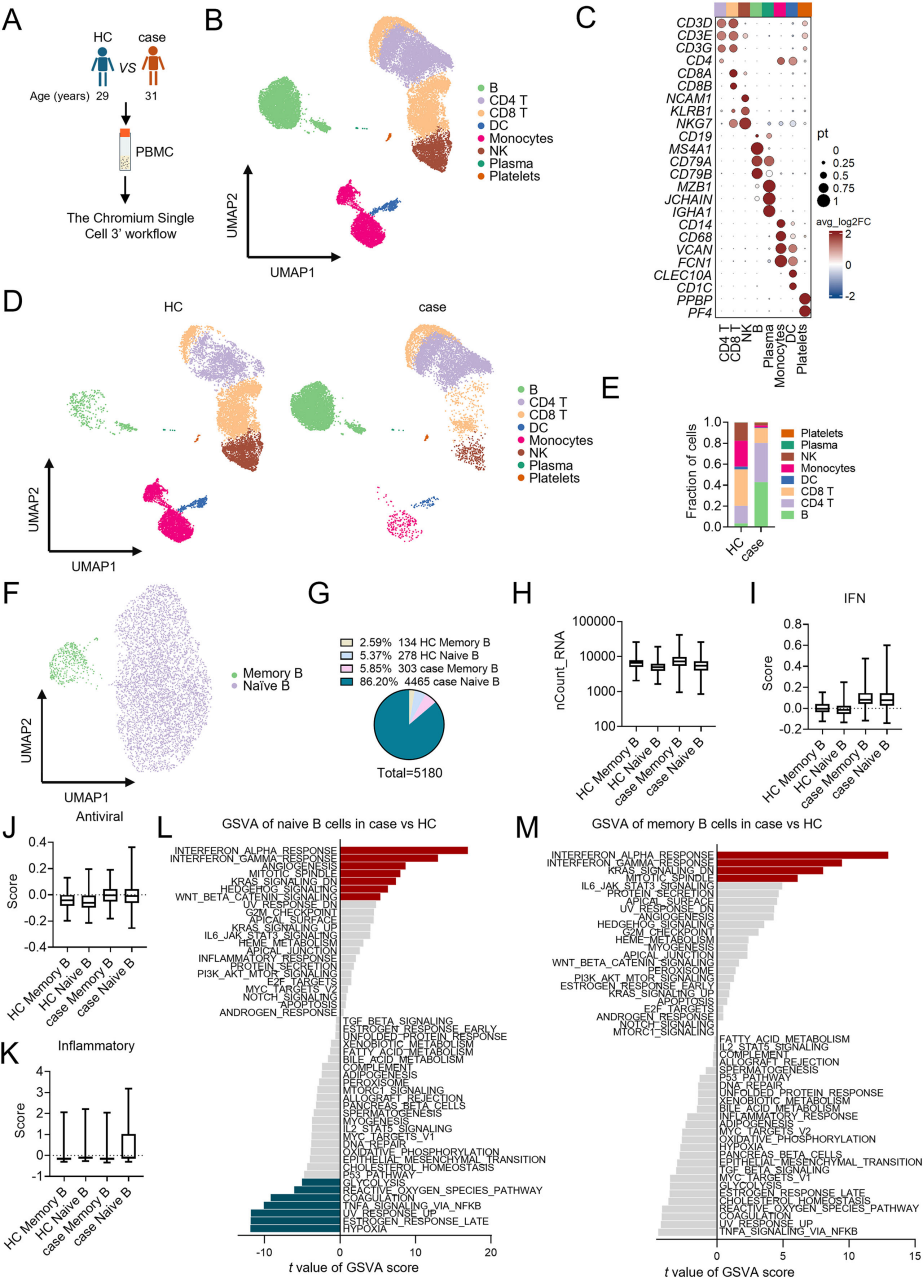
Cell typing of PBMCs in the case relative to HC through scRNA-seq. **(A)** Flowchart depicting the overall experimental design of this study. **(B)** UMAP plots of the 22,665 cells profiled with each cell color-coded for cell types. **(C)** Dot plots of cell type marker genes. Expression values were normalized and scaled to the averages. **(D)** UMAPs of the eight major cell types split by HC and the case, color-coded by cell types. **(E)** Proportion plots of major cell types in HC and case. **(F)** UMAPs of the two major cell subtypes of B cells. **(G)** Pie chart illustrates the proportion of B cell subtypes in HC and the case. **(H)** Box plots of UMIs detected in HC and the case (with plot center, box and whiskers corresponding to median, interquartile range [IQR] and 1.5 × IQR, respectively; n = 134, 278, 303 and 4465 cells for clusters HC memory B, HC naïve B, case memory B and case naïve B, respectively). **(I–K)** IFN **(I)**, antiviral **(J)**, and inflammatory **(K)** scores in B cell subtypes in HC and case samples. n = 134, 278, 303 and 4465 cells for clusters HC memory B, HC naïve B, case memory B and case naïve B, respectively). Data are mean ± SD. **(L, M)** Differential expression of hallmark pathway gene signatures scored per cell using gene set variation analysis (GSVA) of naïve **(L)** and memory **(M)** B cells in the case versus HC. The data shown are t values from a linear model.

Classic risk factors for CAM include older age and dysphagia (19, 28). In contrast, our 31-year-old patient developed CAM with a rare parotid LEC (18, 20, 29, 30), a malignancy seldom linked to DM. Therefore, to investigate the underlying immunologic features, we characterized the cellular atlas of PBMCs obtained at the time of first recurrent DM diagnosis. PBMCs from an age-matched (29-year-old) female healthy donor were used as a control (**Figure 3A**).

Our high-resolution dataset comprised approximately 140,654,478 transcripts across 23,600 cells, with a median detection of 2,290 genes per cell (**Supplementary Figure S1A**) and a minimum threshold of 550 genes per cell (**Supplementary Figure S1B**). Principal component analysis of the top 3,000 DEGs identified 21 distinct cell clusters (**Supplementary Figure S2A**), which were further annotated against established immune lineages using canonical marker genes (**Supplementary Figure S2B**). Following quality filtering and doublet removal, 22,665 high-quality cells were retained yielding representing all eight major immune cell populations (**Figures 3B, C**; **Supplementary Figure S2C**), with a ranked breakdown into 6,088 CD4^+^ T cells (26.9%), 5,587 CD8^+^ T cells (24.6%), 5,180 B cells (22.8%), 3,079 monocytes (13.6%), 2,264 NK cells (10.0%), 366 dendritic cells (DC,1.6%), 36 plasma cells (0.2%), and 65 platelets (0.3%).

A comparative analysis of overall immune cell frequencies identified a significant compositional shift in the case compared to HC. This shift was characterized by a relative expansion of B cells and CD4^+^ T cells, concurrent with a contraction of cytotoxic subsets (CD8^+^ T cells, NK cells) and monocytes (**Figures 3D, E**). To determine if these proportional changes reflected true changes in absolute cell numbers, we performed flow cytometry analysis of lymphocyte subsets, referencing clinical complete blood count (CBC) data and a 40-sample healthy cohort (**Table 1**). Flow cytometry analysis confirmed pronounced lymphopenia at both the initial diagnosis and a 10-month follow-up, with total lymphocyte counts below the healthy reference range (**Table 1**). The absolute numbers of T cells and NK cells were substantially reduced. While the absolute count of B cells is within the reference range. This indicates that the elevated B cell frequency in the scRNA-seq data was a relative increase, attributable to the more severe depletion of other lymphocyte populations (**Table 1**). The subsequent decline in absolute B cell counts during persistent disease suggests that the initial relative abundance may have been a maintenance effect of EBV infection, though EBV DNA was undetectable at the time of sample collection (31, 32).

**Table 1:** Lymphocyte subtypes analysis of the case.

	Control cohort (n=40) mean ± SD		Case (Dec 2024)		Case (Oct 2025)	
**Age, years**	44.6 ± 6.2		31		32	
**Monocytes** **(CBC,** cells/ul)	399 ± 116		430		470	
**Lymphocyte (CBC,** cells/ul)	1935 ± 423		**531↓**		**356↓**	
Lymphocyte subtypes	Count, cells/ul	% of lymphocyte	Count, cells/ul	% of lymphocyte	Counts, cells/ul	%of lymphocyte
CD4 T cells (FC, CD3+CD4+)	729 ± 213	37.75 ± 7.58	**169↓**	31.83	**165↓**	**46.35↑**
CD8 T cells (FC, CD3+CD8+)	520 ± 188	26.69 ± 7.27	**155↓**	29.19	**108↓**	30.34
B cells (FC, CD45+CD19+)	216 ± 87	11.20 ± 3.67	144	**27.12↑**	**52↓**	14.61
NK cells (FC, CD3-CD56+ CD16+)	327 ± 208	16.84 ± 9.75	**63↓**	11.86	**31↓**	8.71

CBC, complete blood cell; FC, flow cytometry. Case numbers are highlighted in bold when their values fall outside the control cohort’s mean ± 1 ×standard deviation.

While EBV can transform B cells through T cell-independent mechanisms (31), the role of B cells in this case of DM was unclear. Unsupervised clustering identified six distinct B cell sub-populations (**Supplementary Figure S2E**). Annotation with canonical markers revealed that the major population consisted of patient-derived naïve B cells (**Figures 3F, G**; **Supplementary Figures S2E–G**). These naïve B cells exhibited transcriptional levels comparable to other B cell subtypes (**Figure 3H**). Pathway enrichment analysis showed that although interferon (IFN) and antiviral response genes were active in the patient’s B cells, the inflammatory response was uniquely and predominantly elevated in the naïve B cell subset (**Figures 3I–K**). In contrast, cytokine and chemokine signaling showed a slight declining trend in the patient’s B cells (**Supplementary Figure S2H**) (33, 34). This suggests that the patient’s naïve B cells contribute to DM pathology may through a specific inflammatory program, rather than through broad antiviral, IFN, or cytokine-mediated activation.

To investigate the basis for naive B cell expansion, we performed gene set variation analysis (GSVA) using hallmark pathway gene signatures. This revealed significant enrichment in the case for proliferative pathways, including mitotic spindle, hedgehog signaling and WNT/β-catenin signaling (**Figure 3L**). Additionally, interferon pathways were more active in patient-derived naive B cells (**Figures 3I, L**). These proliferative and inflammatory characteristics were also observed in memory B cells (**Figure 3M**).

### Whole-exome sequencing

Given the established association between EBV and LEC (7, 35), we performed germline whole-exome sequencing (WES) on peripheral blood to identify potential driver genes or molecular mechanisms underlying DM recurrence. Sequencing generated 145,544,140 total reads, with a mean coverage depth exceeding 200×. We identified 98,002 single-nucleotide variants (SNVs) and 16,872 short insertions and deletions (indels). Of these, 24.04% of SNVs were exonic, and 47.24% of these SNVs were nonsynonymous (**Supplementary Figures S3A, B**). Among the indels, 4.12% were located in exonic regions, and 29.96% of these indels resulted in frameshifts (**Supplementary Figure S3C, D**). Putatively deleterious mutations are summarized in Supplementary Data S1. From the 338 genes harboring nonsynonymous exonic SNVs, we identified 29 genes implicated in immune regulation pathways (**Supplementary Table S1**). None of these genes have been previously reported as known susceptibility genes for DM. However, *Complement C3d Receptor 2* (*CR2*, encode CD21 protein), which functions as a receptor for EBV binding on B and T lymphocytes, has been reported that rs1876453 in this gene are associated with susceptibility to systemic lupus erythematosus SLE (36). In our case, rs367567954 in CR2 is functional unknown but makes the amino acid changed and putatively deleterious.

## Discussion

DM is linked to an increased risk of malignancy compared to the general population, with patients diagnosed with CAM experiencing significantly poorer survival outcomes. Older age is an established clinical risk factor for CAM (19, 28). However, we present a rare case of a young male in his 30s with anti-TIF1γ-positive DM coexisting with parotid LEC. The patient’s LEC was successfully controlled with a timely combination of surgery, radiation, chemotherapy, and immunotherapy. He achieved a complete response (CR) that was maintained one year later, shifting clinical priority from cancer mortality to long-term surveillance. Following an initial improvement in dysphagia and muscle weakness, cyclophosphamide was discontinued in December 2024, and maintenance therapy with methylprednisolone was continued. While this controlled the muscle symptoms, the skin rash persisted. In May 2025, the rash intensified, though no other DM symptoms recurred. Numerous adjustments to his immunosuppressive regimen failed to improve the rash, which was less responsive than in typical DM cases (37–39). The effective management of critical cancer initially directed attention away from the persistent DM. In retrospect, it is uncertain whether maintaining a more aggressive immunosuppressive regimen (continued cyclophosphamide until the resolution of the rash) after December 2024 would have prevented the rash exacerbation.

EBV is not only a key environmental trigger for autoimmune diseases like systemic lupus erythematosus (SLE) and multiple sclerosis (40, 41), but also an oncogenic virus strongly associated with LEC. Given the detection of EBV in both tumor tissue and serology, we consider the LEC to be an EBV-associated malignancy. Though the initial diagnosis of DM three years prior to the discovery of LEC complicates the relationship, the CR efficacy of LEC did not improve the recurrence of DM. This sequence challenges the classical paradigm of CAM, in which malignancy is presumed to induce the myositis (19). Given the immune checkpoint inhibitors (ICIs) are known to cause various immune-related adverse events (irAEs), we could not deny a potential link between toripalimab and the DM flare. However, this case presents atypically: the severe flare, characterized by profound muscle weakness, occurred six months post-treatment, whereas classic ICI-induced myositis typically manifests during therapy (42, 43). The earlier, mild rash during LEC treatment was not the primary concern. While a delayed effect remains possible, the timeline suggests this may not be a classical irAE. But the coexisting of postoperative parotid LEC limited the reign of DM treatment. The patient’s presentation, including anti-TIF1γ positivity, firmly supports a diagnosis of DM. Notably, the transient expansion of activated B-cell populations during disease recurrence may represent a delayed immunologic phenomenon stemming from EBV activation, as it resolved after ten months.

Following the DM recurrence, the patient reported considerable anxiety. The confirmation of a stable head and neck MRI and the resolution of systemic symptoms (dysphagia, muscle weakness) provided relief, leading him to initially disregard the persistent rash. Now, as a young man in his early 30s with a well-controlled LEC, he is actively seeking solutions for rash clearance. Consequently, improving cutaneous disease control has become a critical therapeutic objective. Given that the patient has reached the maximum cumulative dose of effective cyclophosphamide and has shown inadequate response to both methotrexate and tofacitinib, we have decided to initiate rituximab. This choice is also preferred over continued tofacitinib due to the latter’s potential oncogenic risk.

The somatic mutation status of TRIM33 (which encodes TIF1γ) in the LEC tissue is unknown, as the sample was archived at an outside hospital and unavailable for testing. Therefore, we cannot exclude the potential driver role of the LEC in the recurrent DM.

While immunotherapy for the LEC may have boosted systemic immune activation, the DM nonetheless followed an unfavorable course despite effective local tumor control. Furthermore, most DM treatments, particularly immunosuppressants, carry a risk of promoting malignant relapse (44). This created a significant therapeutic challenge in managing the persistent autoimmune disease following the cancer diagnosis. Considering his family responsibilities, the patient stated a preference for an economical treatment option to control his rash and also manage his daily life.

## Conclusion

The co-occurrence of DM and parotid LEC in a young patient presents a complex clinical scenario. The cancer history is a critical determinant in DM treatment planning, restricting therapeutic options due to the risk of immunosuppression. This case emphasizes that such rare associations demand long-term vigilance and highly personalized management strategies to optimize outcomes.

## Data Availability

The datasets presented in this study can be found in online repositories. The names of the repository/repositories and accession number(s) can be found in the article/**Supplementary Material**.
