# Supplementary material for: Recurrent anti-TIF1γ-positive dermatomyositis coexisting with postoperative parotid lymphoepithelial carcinoma: a case report with pathogenesis analysis
Source: Front Immunol. 2026 Feb 16;17:1748650. doi: 10.3389/fimmu.2026.1748650 (PMC12950755; doi:10.3389/fimmu.2026.1748650)
Supplement: Supplementary file 1 [file DataSheet1.pdf]

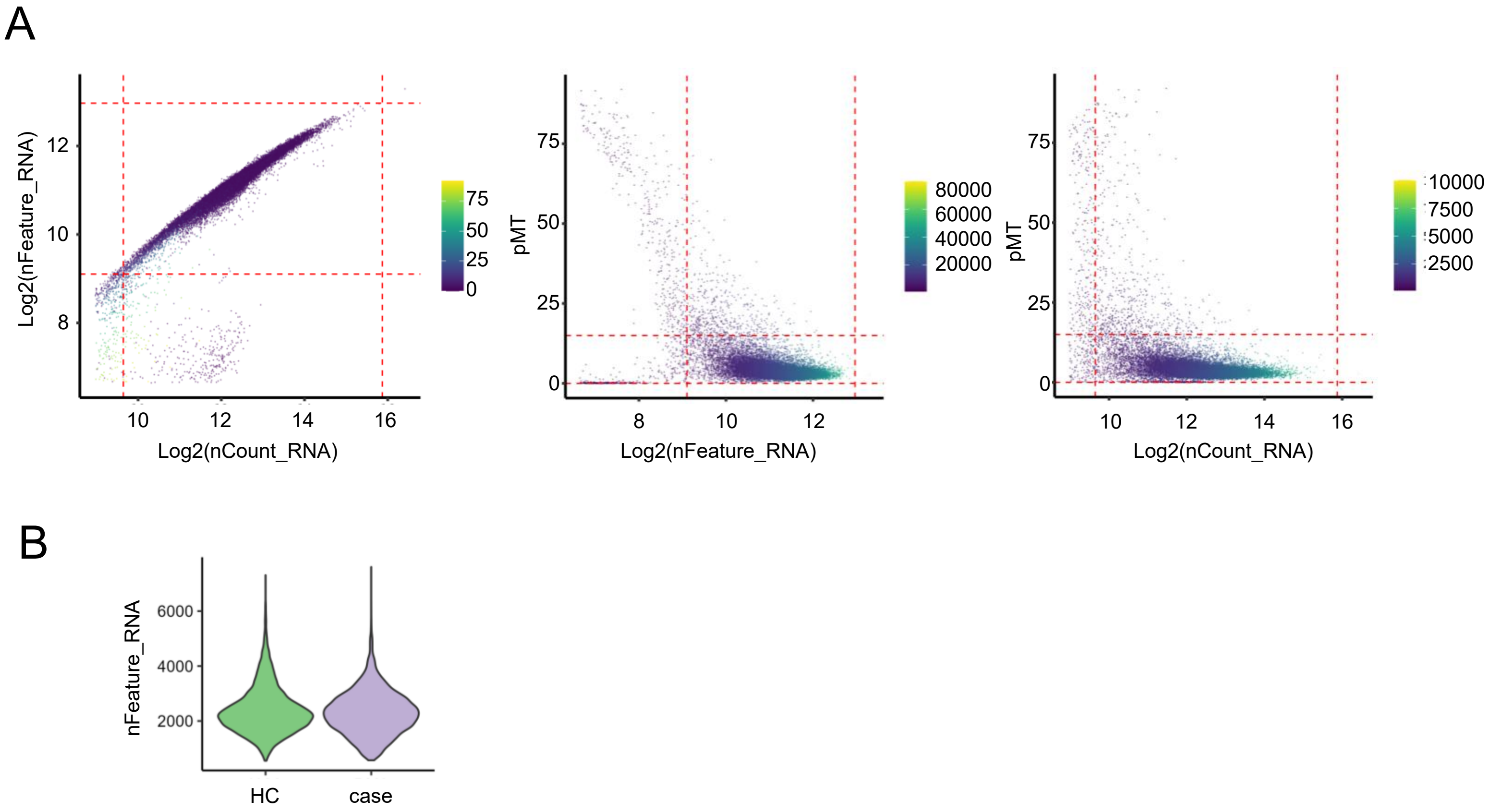

Figure S1. Quality control of scRNA-seq data. A) Scatter plots of quality control parameters of all single-cell transcriptomes analysed in the study, with red dashed lines indicating cut-off values for filtering of high-quality transcripts. **B**) Violin plots showing the distribution of gene counts per cell in healthy control (HC) and case. Data are with the following cell numbers: HC: n = 11,976 cells; case: n = 11,624 cells.

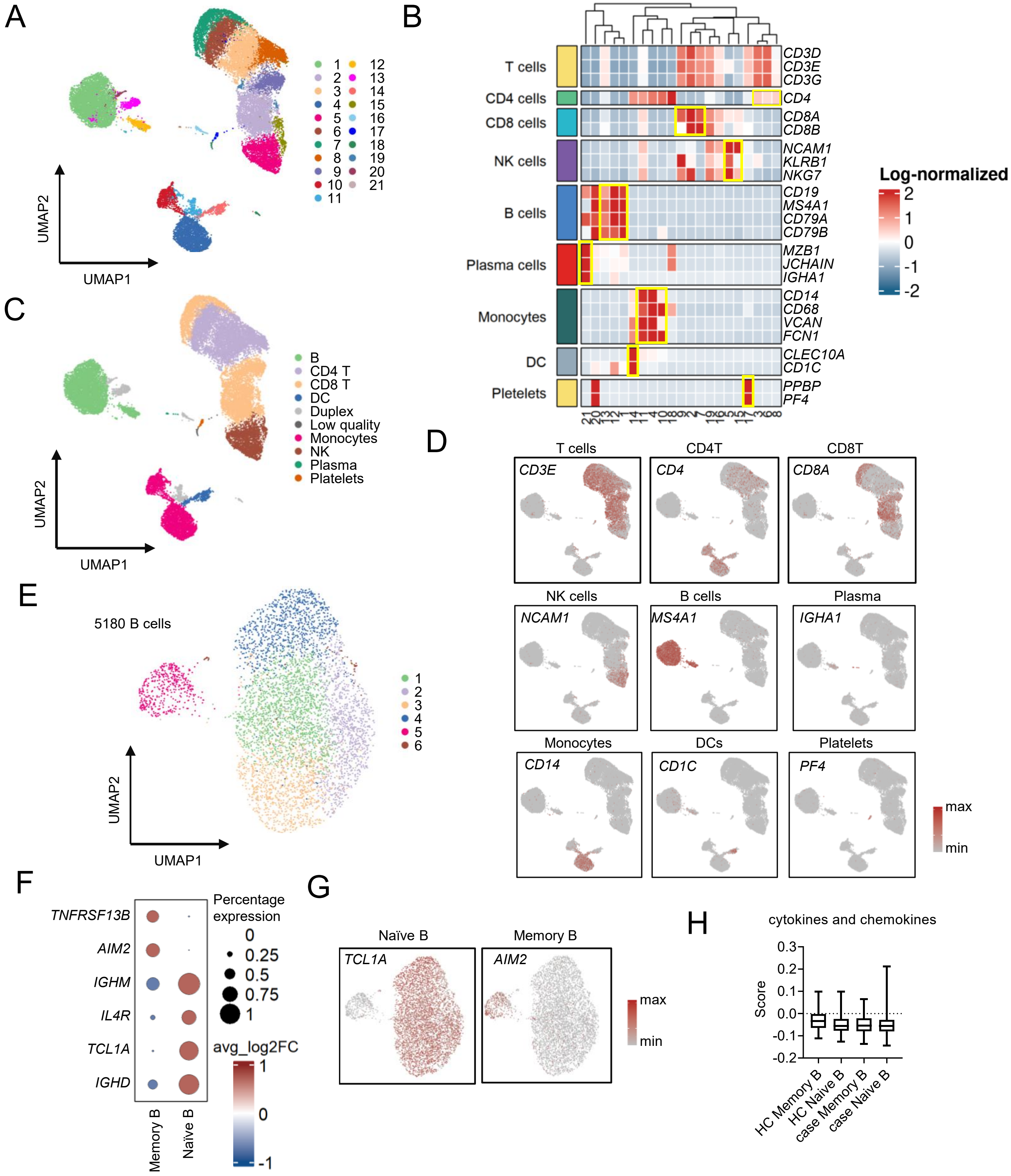

Figure S2. Cell typing of PBMCs in case relative to HC tissues through scRNA-seq. A) UMAP plots of the 21 clusters with each cell color-coded for the associated cluster. **B**) Heatmap illustrating the classical markers of different cell types. Matched clusters are highlighted in yellow. **C**) UMAP plots of the 23,600 cells profiled with each cell color-coded for cell types. Duplex and cells with low quality are also shown. **D**) UMAP plots of all the cells with each cell color-coded for the expression of a marker gene for the individual cell subtypes. **E**) UMAP plots of B cell subtypes with each cell color-coded for the associated subtypes. **F**) Dot plots of B cell subtype marker genes. Expression values were normalised and scaled to the averages. **G**) UMAP plots of the B cells with each subtype color-coded for the expression of a marker gene for the individual cell subtypes. **H**) cytokine and chemokine scores in B cell subtypes in HC and case sampes. n = 134, 278, 303 and 4465 cells for clusters HC memory B, HC naïve B, case memory B and case naïve B, respectively). Data are mean±SD.

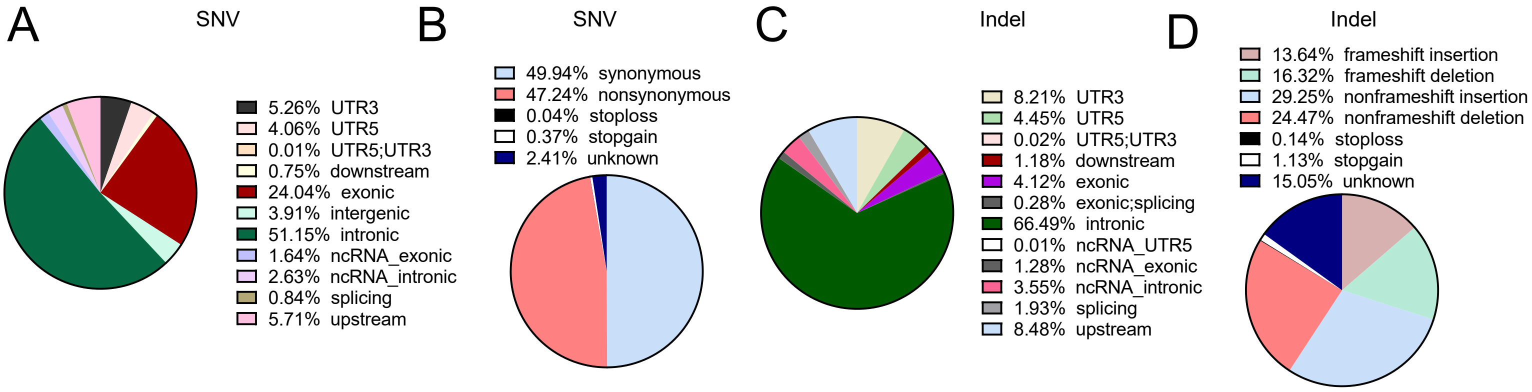

Figure S3. Genomic mutation summary (Germline). A&B) Pie charts depicting distribution (A) and predicted consequences (B) of SNVs in the case. **C&D)** Pie charts depicting distribution (C) and predicted consequences (D) of indels in the case.

Table S1 Genomic mutation summary (germline).

Gene	Mutation site	variant	dbSNP	GO pathway
ABCC9	NM 005691: c.G4564A: p.G1522S	0.429412		GO:0051607
USP17L2	NM 201402:c.C528G: p.C176W	0.444444	rs569397331	GO:0006955, GO:0051607
IFIH1	NM 022168: c.A1041C: p.L347F	0.449074	rs183412282	GO:0006955, GO:0045087, GO:0051607
TIRAP	NM 148910: c.G671A: p.G224E	0.547739	rs758642937	GO:0006955, GO:0045087, GO:0042742, GO:0002694
IFIT5	NM 012420: c.C1084T: p.R362W	0.548387	rs139979024	GO:0006955, GO:0045087, GO:0051607
DEFB116	NM 001037731: c.C124G: p.L42V	0.554455	rs144973782	GO:0006955, GO:0045087, GO:0042742
SYK	NM 003177: c.G1769A: p.R590Q	0.564935	rs748067972	GO:0006955, GO:0045087, GO:0042742, GO:0002250, GO:0050853, GO:0002694
VAMP7	NM 001185183: c.A682G: p.R228G	0.643411	rs192817973	GO:0006955, GO:0045087, GO:0002694
MLH1	NM 000249: c.C649T: p.R217C,	0.488688	rs4986984	GO:0006955, GO:0002250, GO:0002694
FCER2	NM 002002: c.C794G: p.S265C	0.535211	rs117396061	GO:0006955, GO:0002250
NLRP2	NM 017852: c.C1138T: p.R380C,	0.540541	rs189403101	GO:0006955, GO:0045087
CYP26B1	NM 019885: c.C589A: p.L197M	0.436047	rs779832742	GO:0002694
CR2	NM 001877: c.T1051C: p.C351R	0.530973	rs367567954	GO:0006955, GO:0045087, GO:0002250
COLEC11	NM 024027: c.C169T: p.R57W,	0.59375	rs148207876	GO:0006955, GO:0045087
DHX58	NM 024119: c.C1000T: p.R334C	0.453125	rs76998797	GO:0006955, GO:0045087, GO:0051607
HLA-C	NM 002117: c.T992A: p.M331K	0.672727	rs41542414	GO:0006955, GO:0045087, GO:0002250, GO:0071357
C7	NM 000587: c.A1258C: p.K420Q	0.459677	rs3792646	GO:0006955, GO:0045087, GO:0051607
KLRF2	NM 001190765: c.C43T: p.R15C	0.438889	rs74614163	GO:0006955, GO:0045087
STAB1	NM 015136 :c.G4210A: p.V1404M	0.48366	rs201671103	GO:0042742
PKHD1L1	NM 177531: c.G4909C: p.V1637L	0.478261	rs116863919	GO:0006955
DMBT1	NM 004406: c.G5276A: p.G1759D,	0.546099	rs557026873	GO:0006955, GO:0045087, GO:0051607, GO:0042742
ERMAP	NM 018538 :c.A976G: p.N326D,	0.475676	rs151336893	GO:0006955
CDC42EP4	NM 012121: c.A815T: p.D272V	0.536145		GO:0006955, GO:0045087
CR1	NM 000573:c.T2894C: p.I965T,	0.644444		GO:0006955, GO:0045087, GO:0002250, GO:0002694
NCF1	NM 000265: c.G269A: p.R90H	0.44898	rs201802880	GO:0006955, GO:0045087
IL27RA	NM 004843: c.G1574T: p.W525L	0.442177	rs201942765	GO:0006955, GO:0042742, GO:0002250, GO:0002694
IL1RL1	NM 003856: c.C239T: p.A80V,	0.571429	rs34210856	GO:0006955, GO:0002250, GO:0002694
EMILIN1	NM 007046: c.G2371C: p.G791R	0.458599	rs749502229	GO:0042742
SAMSN1	NM 022136: c.G292A: p.G98R,	0.491935	rs545336185	GO:0006955, GO:0002250, GO:0002694

GO:0006955: immune response; GO:0045087: innate immune response; GO:0051607: defense response to virus; GO:0042742: defense response to bacterium; GO:0002250: adaptive immune response; GO:0050853: B cell receptor signaling pathway; GO:0071357: cellular response to type I interferon; GO:0002694: regulation of leukocyte activation

Methods

Single-cell RNA sequencing and Data Analysis

Sample Collection and Processing. Whole blood samples were collected from healthy volunteer and the patient with anti-TIF1γ-positive dermatomyositis. Peripheral blood mononuclear cells (PBMCs) were isolated by density gradient centrifugation and cryopreserved in 90% fetal bovine serum (FBS) and 10% dimethyl sulfoxide (DMSO) using a controlled-rate freezer. Both samples were subsequently processed by Shanghai OE Biotech Co., Ltd., where they underwent erythrocyte lysis and quality control prior to library preparation.

Library Preparation and Sequencing. Single-cell RNA sequencing libraries were constructed using the 10× Genomics Chromium Next GEM Single Cell 3' Reagent Kit (v3.1) according to the manufacturer's instructions. Briefly, single-cell suspensions were loaded onto a Chromium Single-Cell Controller to generate gel bead-in-emulsions (GEMs). Within the GEMs, barcoded cDNA was synthesized via reverse transcription. Following emulsion breakdown and cDNA purification with MyOne Silane Beads, the cDNA was amplified by PCR. The amplified product was then fragmented, end-repaired, A-tailed, and ligated to sequencing adapters to construct the final libraries. Libraries were sequenced on an Illumina NovaSeq 6000 platform to generate 150 bp paired-end reads. The raw sequencing data have been deposited in the Genome Sequence Archive at the National Genomics Data Center under accession number GSA-Human: HRA012299 and are publicly accessible at https://ngdc.cncb.ac.cn/gsa-human (1, 2).

Data Preprocessing and Quality Control. Raw sequencing data were demultiplexed, aligned to the GRCh38 human reference genome, and quantified using the Cell Ranger software pipeline (v9.0.1). The resulting raw gene expression matrix was imported into Seurat (v5.1.0) for downstream analysis in R. We applied stringent quality control filters to remove low-quality cells and potential doublets, excluding cells with: Fewer than 800 or more than 45,000 unique molecular identifiers (UMIs); Fewer than 550 or more than 8,000 detected genes; Mitochondrial gene content exceeding 15% (Figure S1A).

Normalization, Integration, and Clustering. The filtered UMI count matrix was log-normalized using the NormalizeData function. To mitigate batch effects arising from individual donors, we integrated the data using the harmony algorithm (3), with patient ID as a covariate. Prior to integration, the top 3,000 highly variable genes were identified (FindVariableFeatures) to compute integration anchors. Principal component analysis (PCA) was performed on the integrated data. We then constructed a shared nearest-neighbor graph based on the top principal components and performed graph-based clustering using the FindClusters function. Two-dimensional visualization was achieved using uniform manifold approximation and projection (UMAP). Gene expression distributions were visualized using violin plots generated with the VlnPlot function, which employs kernel density estimation (default 'nrd0' method) to determine bandwidth. Due to the large dataset size, individual data points were omitted from these plots to emphasize overall distribution trends.

Inflammatory score, IFN score, antiviral score and cytokine and chemokines score

Scores were calculated using the AddModuleScore function in the Seurat package. Inflammatory score, IFN score, antiviral score and cytokine score were calculated according to published literature (3, 4).

Flow cytometric analyses

Peripheral blood samples were collected and stored in EDTA anticoagulant tubes for analysis within 24 hours. The absolute counts of lymphocytes and monocytes were determined using a Complete Blood Count (CBC) performed on a Sysmex Automated Hematology Analyzers (XN-9100). For the quantification of $CD8^+$ T cells, 100 μL of whole blood was stained with 10 μL of the tetraCHROME CD45-FITC/CD4-PE/CD8-ECD/CD3-PC5 Antibody Cocktail for 20 minutes. The samples were then incubated with Optilyse C Lysing Solution (Beckman, A11895) for an additional 15 minutes. To quantify NK and B cells, 100 μL of whole blood was stained with 10 μL of the tetraCHROME CD45-FITC/CD56-PE/CD19-ECD/CD3-PC5 Antibody Cocktail for 20 minutes, followed by a 15-minute incubation with Optilyse C Lysing Solution (Beckman, A11895). Subsequently, the cells were analyzed using a Navios flow cytometer, following established templates. The proportions of $CD8^+$ T cells, NK cells, and B cells within the total PBMCs were calculated accordingly.

Gene set variation analysis

We conducted GSVA utilizing hallmark pathway gene signatures from the Molecular Signatures Database (MSigDB), accessed via the GSEABase package (v1.64.0). To enhance specificity, we refined each pathway-associated gene set by retaining only unique genes and excluding those that were shared among multiple pathways. Pathway activity scores for individual cells were calculated using the default GSVA parameters, and the results were then converted to z-scores for normalization.

Statistics

All statistical analyses were conducted using R (v4.3.3) and GraphPad Prism 9. Box plots were generated using GraphPad Prism 9. Heatmaps were created using the ComplexHeatmap package.

References

1. Chen T, Chen X, Zhang S, Zhu J, Tang B, Wang A, et al. The Genome Sequence Archive Family: Toward Explosive Data Growth and Diverse Data Types. Genomics Proteomics Bioinformatics. 2021;19(4):578-83.
2. Database Resources of the National Genomics Data Center, China National Center for Bioinformation in 2022. Nucleic Acids Res. 2022;50(D1):D27-d38.

3. Shi J, Pei X, Peng J, Wu C, Lv Y, Wang X, et al. Monocyte–macrophage dynamics as key in disparate lung and peripheral immune responses in severe anti-melanoma differentiation-associated gene 5-positive dermatomyositis-related interstitial lung disease. Clinical and Translational Medicine. 2025;15(2):e70226.
4. He J, Liu Z, Cao Y, Zhang X, Yi C, Zhou Y, et al. Single-cell landscape of peripheral immune response in patients with anti-melanoma differentiation-associated gene 5 dermatomyositis. Rheumatology (Oxford). 2024;63(8):2284-94.
